# Enhanced sensitivity of lateral flow assay for NGAL detection using cysteamine-modified gold nanoparticles

**DOI:** 10.1039/d5ra03287c

**Published:** 2025-11-13

**Authors:** Paweena Tunakhun, Sawinee Ngernpimai, Patcharaporn Tippayawat, Kiattawee Choowongkomon, Sirirat Anutrakulchai, Nicha Charoensri, Ratree Tavichakorntrakool, Sakda Daduang, Oranee Srichaiyapol, Pornsuda Maraming, Jeerati Prompipak, Patcharee Boonsiri, Jureerut Daduang

**Affiliations:** a Centre for Research and Development of Medical Diagnostic Laboratories (CMDL), Faculty of Associated Medical Sciences, Khon Kaen University Khon Kaen 40002 Thailand Jurpoo@kku.ac.th; b Department of Biochemistry, Faculty of Science, Khon Kaen University Khon Kaen 40002 Thailand; c Department of Biochemistry, Faculty of Science, Kasetsart University Bangkok 10900 Thailand; d Department of Medicine, Faculty of Medicine, Khon Kaen University Khon Kaen 40002 Thailand; e Division of Pharmacognosy and Toxicology, Faculty of Pharmaceutical Sciences, Khon Kaen University Khon Kaen 40002 Thailand; f Center for Innovation and Standard for Medical Technology and Physical Therapy (CISMaP), Faculty of Associated Medical Sciences, Khon Kaen University Khon Kaen 40002 Thailand; g Department of Biochemistry, Faculty of Medicine, Khon Kaen University Khon Kaen 40002 Thailand

## Abstract

Neutrophil gelatinase-associated lipocalin (NGAL) has emerged as a crucial biomarker for various clinical conditions, including acute kidney injury. However, enhancing the sensitivity of the lateral flow strip (LFS) for NGAL detection remains a challenge. LFS has revolutionized point-of-care diagnostics, offering rapid and cost-effective detection of biomarkers. This study explores the potential of cysteamine gold nanoparticles (AuNPs) as a novel conjugate for NGAL detection in LFS, comparing their sensitivity enhancement against traditional citrate-coated AuNPs (Cit-AuNPs). Through comprehensive experimentation and analysis, we demonstrate the superior performance of cysteamine-coated AuNPs (Cys-AuNPs) LFS in detecting NGAL, paving the way for more sensitive point-of-care diagnostics in relevant clinical settings.

## Abstract 2

An aggregation-based assay was developed to assess monoclonal antibody orientation on gold nanoparticles (AuNPs) for NGAL detection. Cysteamine-coated AuNPs showed better antibody orientation than citrate-coated AuNPs, improving NGAL LFS sensitivity.

## Graphical abstract

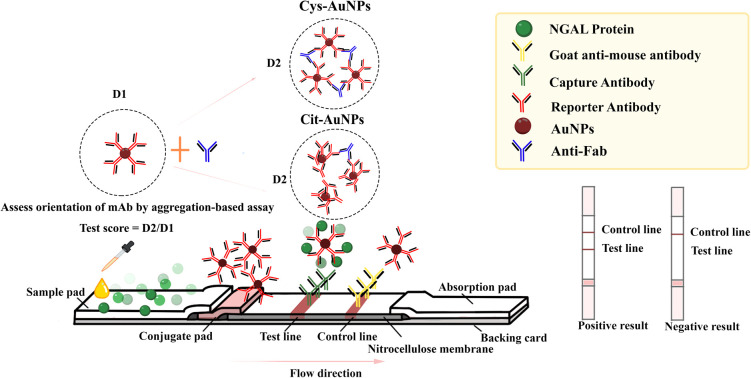

## 1 Introduction

Acute kidney injury (AKI) is a prevalent and serious condition characterized by a rapid decline in kidney function, leading to the accumulation of waste products and disturbances in fluid and electrolyte balance.^1^ Globally, approximately 21.6% of adults and 33.7% of children experience AKI, with higher rates observed in critical care settings and after cardiac surgery.^2^ The mortality rate for patients requiring renal replacement therapy is alarmingly high, reaching up to 50%.^3^ Early detection and intervention are crucial to mitigate the progression of kidney disease and reduce associated morbidity and mortality.

Neutrophil gelatinase-associated lipocalin (NGAL) has emerged as a vital biomarker for AKI due to its rapid elevation in response to kidney injury.^4^ The lateral flow strip (LFS) are chosen for NGAL detection in this study due to their simplicity, rapidity, and cost-effectiveness, making them invaluable tools for point-of-care diagnostics.^5^ These assays enable timely intervention and treatment by quickly detecting biomarkers associated with various clinical conditions.

One promising approach involves the use of gold nanoparticles (AuNPs) as labels for signal amplification. AuNPs offer several advantages, including ease of functionalization, biocompatibility, and optical properties suitable for detection.^6^ In traditional LFS, citrate-stabilized AuNPs are often used due to their stability and ease of synthesis.^7^ However, the sensitivity of AuNP-based assays is strongly influenced by the surface chemistry used to immobilize antibodies. Traditional citrate-capped AuNPs (Cit-AuNPs) primarily rely on weak electrostatic adsorption, which often results in random antibody orientation and reduced antigen accessibility.^8^ In contrast, cysteamine-stabilized AuNPs (Cys-AuNPs) provide a more versatile platform for bioconjugation. Cysteamine is a bifunctional linker molecule that contains both a thiol (–SH) and a primary amine (–NH_2_) group. The thiol moiety enables strong and stable Au–S coordination to the nanoparticle surface, while the terminal amine group can interact with biomolecules.^9^ This bifunctional nature makes cysteamine particularly attractive for biosensor applications. The presence of primary amine groups allows directional immobilization of antibodies *via* interactions with negatively charged carboxylate groups in the Fc region, thereby orienting antigen-binding Fab domains outward and improving target accessibility.^9^

In this study, the use of Cys-AuNPs presents an intriguing opportunity to overcome the sensitivity limitations of traditional LFS for NGAL detection. These unique physicochemical properties make cysteamine a suitable and promising functionalizing agent for improving LFS performance in biomarker detection. By leveraging the distinctive properties of Cys-AuNPs, it may be possible to achieve significant improvements in the sensitivity and accuracy of NGAL detection in LFS.

This study explores the potential of Cys-AuNPs as a novel conjugate for NGAL detection in LFS. It aims to demonstrate their superiority over Cit-AuNPs in terms of sensitivity enhancement. Through a comprehensive investigation, including synthesis optimization, conjugation strategies, and assay performance evaluation, we aim to establish Cys-AuNPs LFS as a next-generation platform for NGAL detection, with implications for improved diagnosis and management of NGAL-associated clinical conditions.

## 2 Method

### 2.1 Reagents and materials

Cysteamine hydrochloride, globulin, bilirubin, hemoglobin, albumin, bovine serum albumin (BSA), human serum albumin (HSA), gold(iii) chloride trihydrate (HAuCl_4_·3H_2_O, 99%), trisodium citrate dihydrate (C_6_H_5_Na_3_O_7_. 2H_2_O), and NGAL ELISA kits were sourced from Sigma-Aldrich (St. Louis, MO, USA). Potassium carbonate (K_2_CO_3_), glucose, and ascorbic acid were procured from KemAus (Cloisters Cherrybrook, Australia). C-terminal polyhistidine-tagged recombinant human lipocalin-2 (NGAL) was supplied by Sino Biological Inc (Beijing, China). Mouse monoclonal antibodies (mAbs) targeting human NGAL (mAb-NGAL) were acquired from Fitzgerald Industries International (cat.10-1575 for the capture antibody and cat.10-1576 for the reporter antibody) located in North Acton, MA, USA. Goat anti-mouse antibodies were obtained from Lampire Biological Laboratories, Inc (Pennsylvania, USA). Nitrocellulose membranes (CN140) were purchased from Sartorius Stedim Biotech SA (Goettingen, S.A., Germany). The backing card, absorbent pad (CF5), untreated conjugated pad, and sample pad (GR470) were provided by Global Life Sciences Solutions USA LLC (Marlborough, MA, USA).

### 2.2 Ethical approval

Clinical samples were sourced from the Clinical Laboratory Unit at Srinagarind Hospital, Khon Kaen University, Thailand. This research received approval from the Khon Kaen University Ethics Committee (HE684002).

### 2.3 Sample collection and eligibility criteria

Participants aged 18 years or older with a confirmed diagnosis of AKI were included in the study, excluding those with a history of chronic kidney disease (CKD). A total of 20 urine samples from AKI patients and 20 from healthy individuals were collected. Each participant underwent a comprehensive medical history review and clinical evaluation. AKI diagnosis was based on the KDIGO clinical practice guidelines, involving the assessment of blood creatinine levels and urine output.

### 2.4 Synthesis and characterization of Cit-AuNPs

The synthesis of Cit-AuNPs with a diameter of 12.7 nm was achieved using a modified citrate reduction method.^10^ The process involved boiling a 1 mM solution of HAuCl_4_·3H_2_O (100 mL) at 250 °C for 10 minutes. Subsequently, 10 mL of a 38.8 mM sodium citrate solution was rapidly introduced to the mixture, followed by an additional 10 minutes of heating. After cooling to ambient temperature, the resulting AuNP suspension was filtered through a 0.45 µm membrane. The synthesized AuNPs were thoroughly characterized using various analytical techniques. Transmission electron microscopy (TEM) images were analyzed using ImageJ software to determine the nanoparticle size distribution. The optical properties of the AuNPs were assessed by UV-Vis spectroscopy, measuring the absorption spectrum between 400–800 nm.^11^ Additionally, dynamic light scattering (DLS) was employed to measure the hydrodynamic diameter of the nanoparticles,^12^ while zeta potential measurements provided information on their surface charge.^13^ These characterization methods collectively offered a comprehensive understanding of the physical and chemical properties of the synthesized Cit-AuNPs.

### 2.5 Preparation of Cys-AuNPs

We adapted an existing protocol to produce stable cationic AuNPs with a consistent size range of 10–20 nm.^14,15^ The process began by adding 40 mL of an aqueous HAuCl_4_ solution (1.4 mM) to a clean flask at room temperature. Under vigorous stirring, 400 µL of an aqueous solution of cysteamine hydrochloride (213 mM) was slowly introduced. After 20 minutes, 40 µL of freshly prepared sodium borohydride (NaBH_4_, 10 mM) was added to the mixture, maintaining the same stirring intensity. The following 8–10 minutes were crucial for precise nanoparticle formation, evident from the appearance of a deep wine-red color. The Cys-AuNPs were subsequently characterized using standard techniques, including TEM, UV-Vis spectroscopy, and zeta potential analysis to assess size and surface charge.

### 2.6 Conjugation of antibodies to AuNPs and optimization

The conjugation process of citrate-capped^16^ and cysteamine-capped AuNPs^17^ with the antibody mAb-NGAL began by optimizing the pH while maintaining a constant antibody concentration of 1.0 mg mL^−1^, following adjustments to a previous protocol. The pH of both Cit-AuNP and Cys-AuNP dispersions was adjusted using a 0.1 M K_2_CO_3_ solution to reach pH levels of 5, 6, 7, and 8, followed by dilution with deionized water (DI) to achieve an optical density (OD) of 1. The conjugation involved adding 12 µL of mAb-NGAL at the fixed 1.0 mg mL^−1^ concentration to 0.8 mL of each AuNP dispersion. The mixture was incubated at room temperature on a rotator for 15 minutes to facilitate the antibody binding to the AuNPs. Afterward, 90 µL of 10% BSA was added to reach a final concentration of 1% BSA, and the samples were incubated for another 10 minutes on a rotator to block non-specific binding. Following incubation, the samples were centrifuged at 6000 g for 20 min to pellet the mAb-NGAL-AuNPs, the supernatant was, and the pellets were redispersed in 200 µL of a suspension solution containing 10 mM phosphate buffer (PB), 5% sucrose, 1% BSA, and 0.25% Tween 20, adjusted to pH 7.4. An aggregation-based assay was conducted to investigate the orientation of mAbs adsorbed onto AuNPs and determine the best pH for conjugation. This involved performing an anti-Fab aggregation assay to semi-quantitatively assess the orientation of the adsorbed mAbs, following a previously established method.^18^ The assay began by measuring the mean hydrodynamic diameter of each conjugate suspension using DLS, recording this initial size as D1 based on the intensity distribution. Then, 50 µg of Fab-specific anti-mouse IgG was added to 100 µL of the conjugates, and the mixture was allowed to react for 1 hour. Afterward, the mean hydrodynamic diameter was measured again and recorded as D2. A test score was then calculated by dividing D2 by D1 (D2/D1). An increase in this score reflects greater aggregation, suggesting that more Fab fragments on the conjugates are accessible. To evaluate the conjugation efficiency of antibodies to AuNPs, we modified the method from a previous study by measuring the concentration of unbound antibodies in the supernatant after centrifugation using a NanoDrop spectrophotometer.^19^ Initially, 12 µL of mAb-NGAL at a concentration of 1.0 mg mL^−1^ was added to each type of AuNP, resulting in a total antibody input of 0.012 mg. After the conjugation process, the samples were centrifuged to separate the AuNP-antibody conjugates from unbound antibodies. The supernatant was carefully collected for further analysis. The concentration of unbound IgG in the supernatant was determined by measuring the absorbance at 280 nm using the NanoDrop spectrophotometer, which allows for direct quantification of protein concentration based on absorbance values. A standard curve was established using absorbance values for known antibody concentrations to determine accurately the amount of unbound IgG in the supernatant. The amount of bound antibodies was calculated by subtracting the concentration of unbound IgG in the supernatant from the initial IgG concentration added during the conjugation process. The following formula was used:Bound Ab (mg) = initial Ab (mg) − unbound Ab (mg)

The percentage of bound antibodies, indicating the conjugation efficiency, was calculated using the formula:% of bound Ab = (bound Ab (mg)/initial Ab (mg)) × 100

To further assess the impact of antibody conjugation, the optical properties of the mAb-NGAL-AuNPs (reporter antibody) were examined using a UV-visible spectrophotometer across the 400–800 nm wavelength range and compared with those of bare AuNPs.^8^ Additionally, zeta potential analysis and hydrodynamic diameter measurements were conducted to evaluate the surface charge and size distribution changes.^20^

### 2.7 Assembly of LFS

**Fig. 1:**
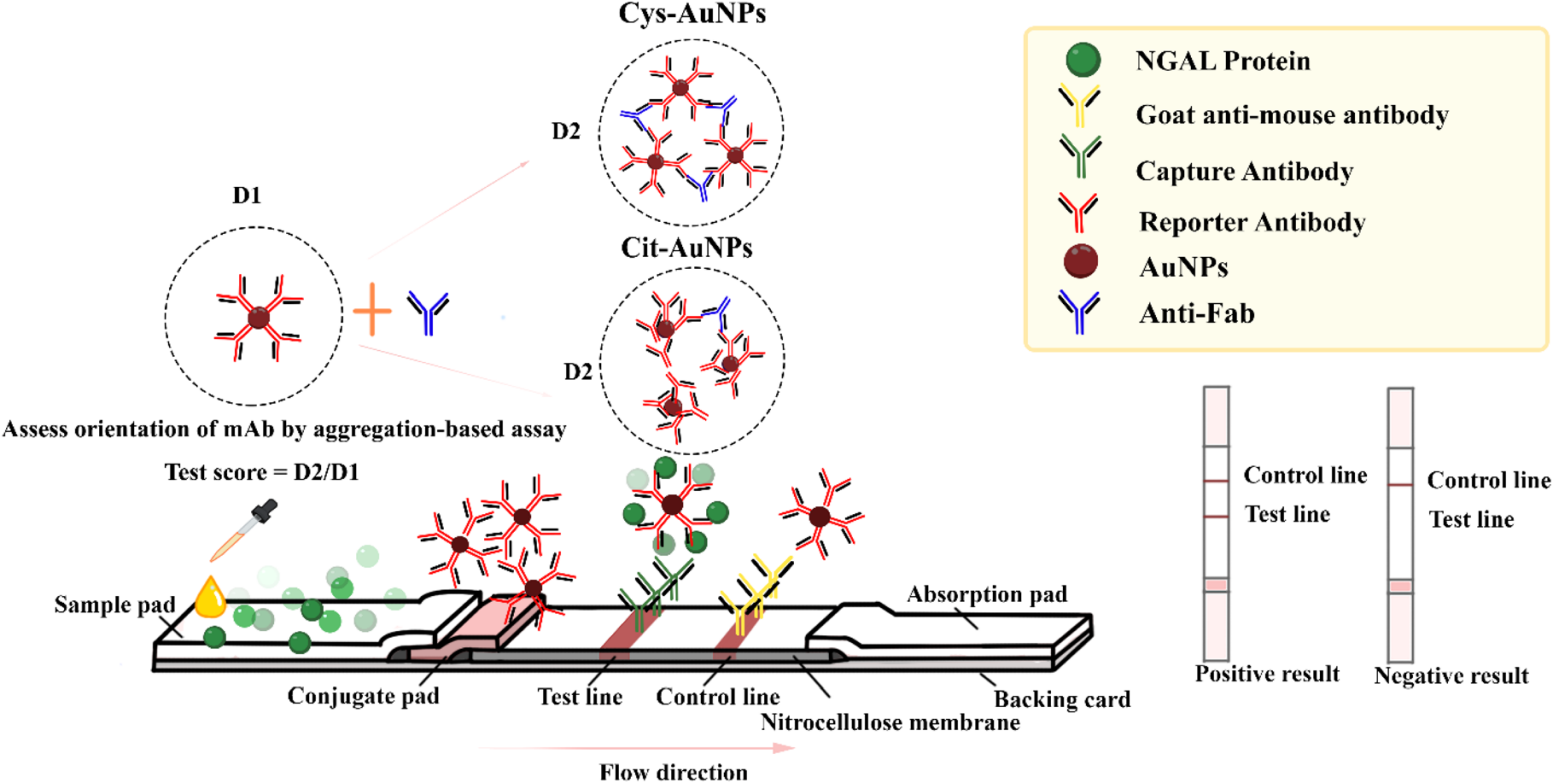
The schematic diagram of the Lateral Flow Strip (LFS) for NGAL detection, utilizing Cys-AuNPs and Cit-AuNPs, and the aggregation test for orientation.

To construct the LFS, five components were assembled: the conjugate pad, nitrocellulose membrane, backing card, absorbent pad, and sample pad (Fig. 1). The assembly of the LFS began by applying the test line (TL), which contained 2 mg mL^−1^ of mAb-NGAL, and the control line (CL), which contained 1 mg mL^−1^ of goat anti-mouse antibody, using the KinBio Platform Dispenser (Shanghai KinbioTech Co., Ltd, Shanghai, China). The dispenser operated at a speed of 50 mm s^−1^, with precise coordinates set for the *X*-axis (vertical position at 18 mm), *Y*-axis (horizontal position at 26 mm), and *Z*-axis (height of the dispensing tip at 16 mm). After the application, the nitrocellulose membrane (CN140) was dried at 37 °C for two hours.

### 2.8 Comparative analysis of sensitivity for LFS using Cys-AuNPs and Cit-AuNPs

To evaluate the sensitivity of the LFS, standard NGAL protein solutions with concentrations ranging from 0 to 75 ng mL^−1^ were prepared. These solutions were diluted in phosphate-buffered saline (PBS) with a pH of 7.4 and supplemented with 0.5% BSA. A 10 µL aliquot of each NGAL concentration was applied to the sample pad of the LFS. This was followed by adding 110 µL of 5 mM phosphate buffer (pH 7.4) containing 1% Triton X-100. The intensity of the test line was then measured using the RapidScan ST5 lateral-flow assay reader (Eurofins Shanghai Co., Ltd). The visual limit of detection (vLOD) was defined as the lowest NGAL concentration that produced a visible purple color on the test line, detectable by the naked eye. Photos of the strips were captured using a RapidScan ST5 LFS reader (Eurofins Shanghai Co., Ltd).

### 2.9 Preparation of conjugate pads

Pre-treatment of conjugate pads with various chemical buffers is essential for achieving optimal release and stability in immune strips.^21^ This study assessed the effectiveness of three distinct buffers, which were prepared according to standard protocols and then applied to the conjugate pads during the pre-treatment process. In the first condition, the pads were left untreated. In the second, the pads were treated with a 10 mM phosphate-buffered (PB) solution at pH 7.4, containing 2% BSA, 2% polyvinylpyrrolidone (PVP), 1% sucrose, and 0.25% Tween 20. The third condition involved treating the pads with a 10 mM phosphate buffer (PB) at pH 7.4, supplemented with 5% sucrose, 1% BSA, and 0.25% Tween 20. These buffers were applied to conjugate pads that had been pre-cut into 4 mm × 10 mm pieces, with 20 µL of buffer added to each pad. The pads were then dried overnight at 37 °C. The following day, 5 µL of the optimized mAb-NGAL-AuNPs solution (as described in Section 2.6) was carefully pipetted onto each pad. After this, the pads were further dried at 37 °C for two hours to stabilize the mAb-NGAL-AuNPs before being integrated into the LFS. This systematic approach allowed for a comprehensive evaluation of the different buffer treatments and their impact on the performance of the immune strips.

### 2.10 Specificity evaluation of NGAL detection by LFS

To determine the specificity of the LFS for NGAL detection, cross-reactivity tests were conducted with various substances that are typically found in urine. The LFS response to 9.38 ng mL^−1^ of NGAL was compared with its response to other substances, including albumin and globulin (both at 10 mg dL^−1^), ascorbic acid (40 mg dL^−1^), bilirubin (4 mg dL^−1^), glucose (500 mg dL^−1^), and hemoglobin (10 mg dL^−1^). The intensity of the resulting bands on the membrane was measured using the RapidScan ST5 reader software to assess potential cross-reactions.

### 2.11 Evaluation of Cys-AuNP conjugate stability in solution and dry states

To evaluate the stability of Cys-AuNPs conjugate with antibodies under different storage conditions, conjugates were prepared under optimal conditions and monitored over time. The study examined two conditions: (1) conjugates stored in solution and (2) conjugates dried onto conjugate pads in LFS. Both conditions were stored at 4 °C throughout the study. For the conjugates in the solution, 100 µL aliquots were dispensed into separate microtubes.

To assess potential aggregation, 5 µL of a 1% (w/v) NaCl solution was added to each well every week for four consecutive weeks. The stability of the conjugates was monitored weekly using UV-Vis spectroscopy.^22^ For the dried conjugates, the conjugate pads embedded within LFS were stored at 4 °C. At each time point, the test strips were evaluated by running 4.69 ng mL^−1^ NGAL solutions and analyzing the signal intensity of the test lines. This approach allowed for the observation of changes in signal response over time, reflecting the stability of the dried conjugates. By tracking the changes under each storage condition, this study provides insights into the long-term stability of Cys-AuNP conjugates in both liquid and dry states.

### 2.12 NGAL detection in clinical samples from AKI and healthy individuals using LFS

The clinical urine samples (*n* = 40) were assessed using the developed LFS. The urine samples, consisting of 20 from AKI patients and 20 from healthy individuals, were analyzed according to the LFS procedure. In this method, urine samples were applied to the LFS, and the presence or absence of a band was observed, indicating the detection of NGAL. AKI was determined based on KDIGO guidelines.

## 3 Results and discussion

### 3.1 Characterization of Cit-AuNPs and Cys-AuNPs

#### 3.1.1 TEM analysis and size distribution

**Fig. 2:**
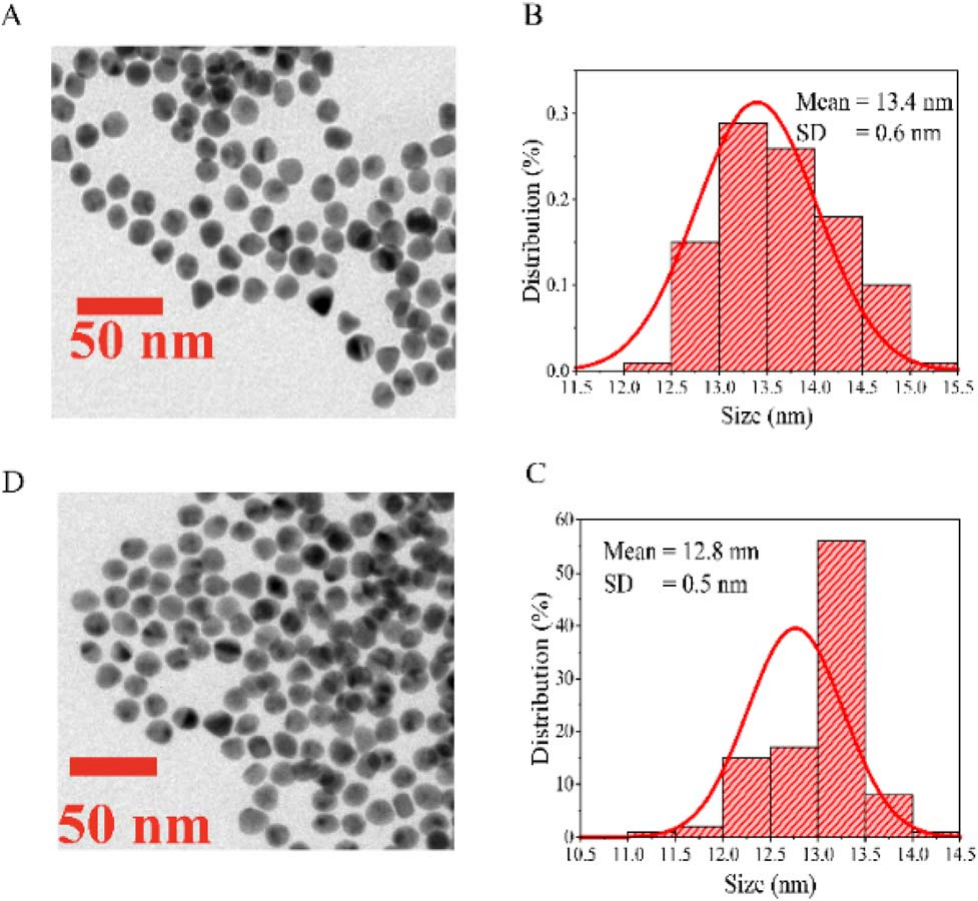
(A and B) TEM images and size distribution histograms of AuNPs: (A) Cit-AuNPs with a diameter of 13.4 ± 0.6 nm, (B) Cys-AuNPs with a diameter of 12.8 ± 0.5 nm; (C and D) UV-Vis spectra of Cit-AuNPs and Cys-AuNPs.

TEM was employed to analyze the diameter morphologies of Cit-AuNPs and Cys-AuNPs. The TEM images showed that Cit-AuNPs had an average diameter of 13.4 nm, while Cys-AuNPs exhibited a slightly smaller average diameter of 12.8 nm. Both types of AuNPs were spherical and well dispersed, as demonstrated in Fig. 2A and B.

#### 3.1.2 UV-vis spectroscopy

**Fig. 3:**
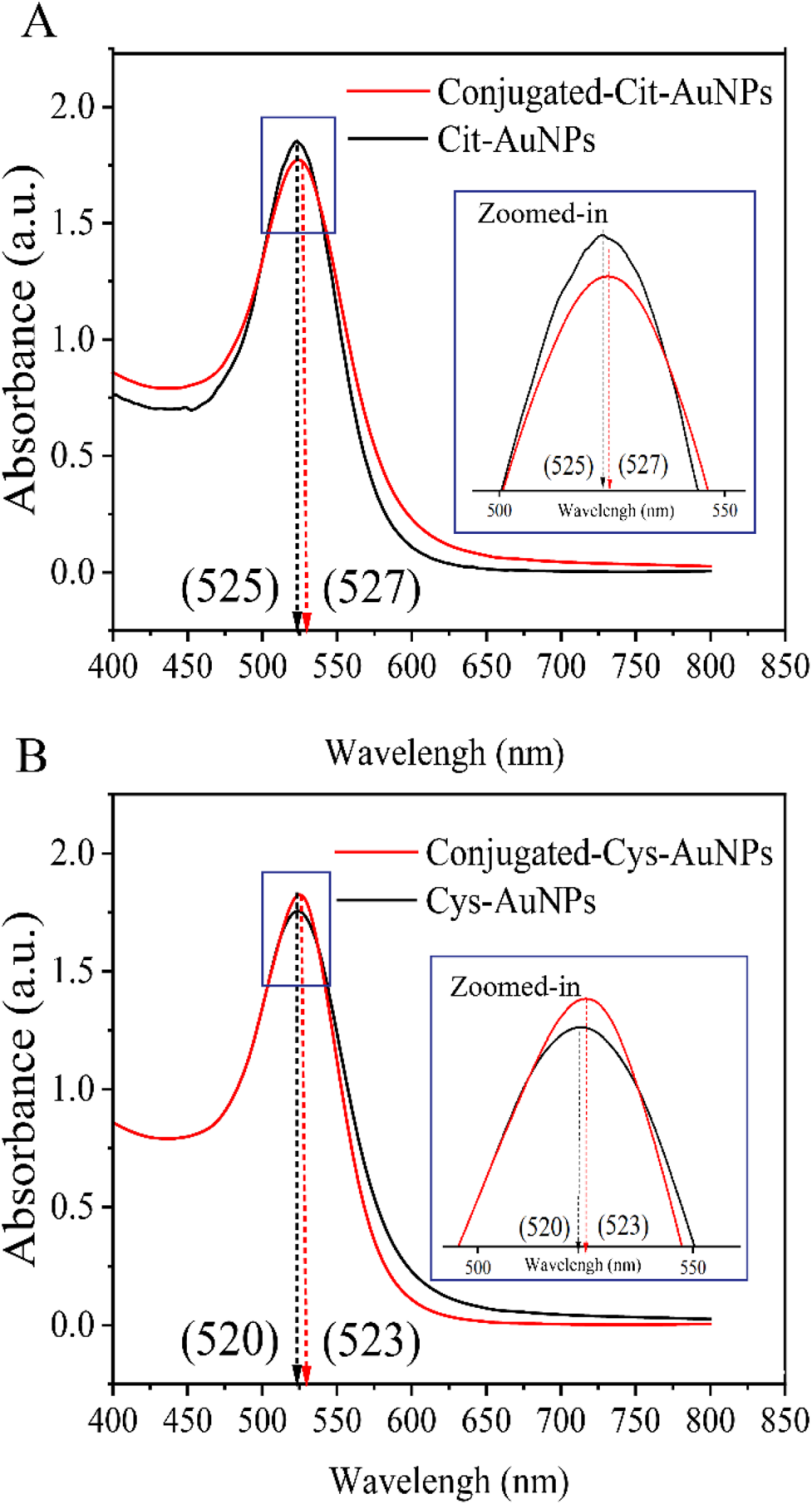
UV-Vis spectroscopy analysis of AuNPs and antibody-conjugated AuNPs (A) UV-Vis spectrum of Cit-AuNPs, displaying a maximum absorbance at 525 nm, which shifts to 527 nm after antibody conjugation. (B) UV-Vis spectrum of Cys-AuNPs, showing a peak at 520 nm, shifting to 523 nm following antibody conjugation.

The optical properties of the AuNPs were investigated using UV-Vis spectroscopy in the wavelength range of 400–800 nm. Cit-AuNPs displayed a maximum absorbance at 525 nm, whereas Cys-AuNPs showed a peak at 520 nm, as seen in Fig. 3A and B. Following the conjugation process with antibodies, a slight red shift in the maximum absorbance wavelengths was observed, shifting to 527 nm for Cit-AuNPs and 523 nm for Cys-AuNPs (Fig. 3A and B). This shift in the plasmon resonance peak suggests effective antibodies binding to the nanoparticle surfaces, likely due to the change in the local refractive index around the AuNPs after antibody conjugation.^23^

#### 3.1.3 Hydrodynamic diameter and zeta potential

Hydrodynamic diameters and zeta potentials were measured to further confirm the surface modification of the AuNPs before and after conjugation with mAbs (conjugated-AuNPs). These results are summarized in Table 1. An increase in hydrodynamic diameter was observed after conjugation, which could be attributed to the larger size of the antibody molecules. Specifically, for Cit-AuNPs, the hydrodynamic diameter increased from 48.61 nm ± 1.18 to 52.66 nm ± 1.28 after conjugation. Similarly, for Cys-AuNPs, the hydrodynamic diameter increased more significantly from 45.25 nm ± 2.80 to 73.09 nm ± 0.64. This substantial increase in size for Cys-AuNPs suggests a higher degree of antibody attachment. Zeta potential measurements indicated a significant change in surface charge upon antibody attachment. For Cit-AuNPs, the zeta potential shifted from −7.73 mV ± 0.26 to −0.47 mV ± 0.04 after conjugation, reflecting a reduction in negative charge. In contrast, Cys-AuNPs showed a decrease in positive charge, from 25.86 mV ± 1.69 to 19.93 mV ± 0.82. These changes in zeta potential further confirm the successful conjugation of antibodies to the AuNPs, as the alteration in surface charge indicates the modified surface chemistry and interaction of antibodies with the AuNP surfaces. The discrepancy between TEM and DLS sizes arises from the fundamental difference in measurement principles. TEM measures the core size of dried particles under vacuum, while DLS reports the hydrodynamic diameter, which includes the solvation layer and any adsorbed molecules (*e.g.*, cysteamine, proteins). The larger DLS size of Cys-AuNPs (∼73 nm) likely reflects the formation of a protein corona and the presence of hydrated layers and surface functional groups.^24^

**Table 1:** Comparison of Cit-AuNPs and Cys-AuNPs characterization *via* DLS and zeta potential measurements

Type of AuNPs	Characterization	Bare AuNPs	Conjugated AuNPs (D1)
Cit-AuNPs	DLS (nm)	48.61 ± 1.18	52.66 ± 1.28
	Zeta potential (mV)	−7.73 ± 0.26	−0.47 ± 0.04
Cys-AuNPs	DLS (nm)	45.25 ± 2.80	73.09 ± 0.64
	Zeta potential (mV)	25.86 ± 1.69	19.93 ± 0.82

#### 3.1.4 FTIR spectra of Cit-AuNPs and Cys-AuNPs

**Fig. 4:**
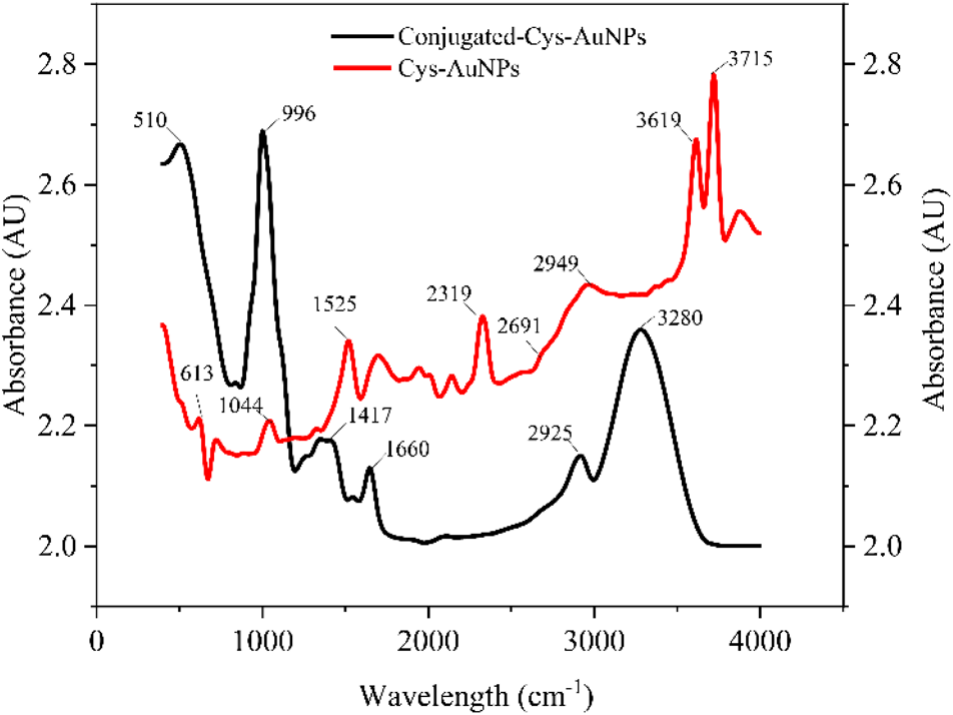
FTIR spectral analysis of Cys-AuNPs and conjugated-Cys-AuNPs The black line represents the spectrum for conjugated-Cys-AuNPs, while the red line represents the spectrum for Cys-AuNPs. Peaks at specific wavenumbers indicate characteristic functional groups.

The FTIR spectra obtained in this study confirm successful conjugation at different functionalization stages (Fig. 4). Comparing Cys-AuNPs and conjugated-Cys-AuNPs reveals distinct spectral shifts that indicate antibody binding. In the Cys-AuNP spectrum, characteristic peaks at 2691 cm^−1^ indicate the presence of disulfide (S–H) groups.^25^ Characteristic peaks at 510 cm^−1^ represent intrinsic disulfide bridges within the antibody structure itself, particularly in the hinge region and variable domains.^26^ The presence of these peaks actually confirms successful antibody immobilization on the Cys-AuNPs, as they indicate that the antibody maintains its native structural integrity upon binding. The distinctive peak shift of the amine (–NH) stretching band from 1525 cm^−1^ to 1660 cm^−1^ and the new peak at 1660 cm^−1^ in the conjugated-Cys-AuNP spectrum represent the intrinsic amide I and amide II bands of the immobilized antibody. These spectral changes indicate successful antibody adsorption and conformational changes upon immobilization. Additionally, the peak at 2319 cm^−1^ may be associated with the interaction of borate residues from the synthesis process with cysteamine. The appearance of a peak at 2949 cm^−1^ corresponds to C–H stretching vibrations, indicating structural stabilization of the nanoparticle conjugate.^27^ The broad peak at 3280 nm, associated with hydrogen bonding or N–H stretching, further supports this interaction, suggesting secondary stabilization *via* electrostatic forces or hydrogen bonding between cysteamine and the antibody.^28^ The primary conjugation mechanism involves non-covalent interactions. Ionic interactions contribute significantly to the stability and orientation of immobilized antibodies. The positively charged amine (–NH_3_^+^) groups in cysteamine can interact electrostatically with the negatively charged carboxyl (–COO^−^) groups of antibodies, providing the primary binding mechanism for antibody immobilization. Additionally, residual borate ions from the synthesis process may contribute to weak ionic interactions with charged regions of the antibody, further supporting the conjugate's stability. The combined ionic interactions and hydrogen bonding energy ensure that antibodies remain in a well-oriented configuration, maximizing antigen recognition. The orientation and stability of immobilized antibodies are crucial for preserving antigen-binding efficiency. The combined ionic and hydrogen bonding energy ensures that antibodies remain in a well-oriented configuration, maximizing antigen recognition. This is particularly advantageous in immunoassays, as it prevents random antibody attachment that could obstruct antigen-binding sites.

### 3.2 Optimization of pH conditions for antibody conjugation to AuNPs

**Fig. 5:**
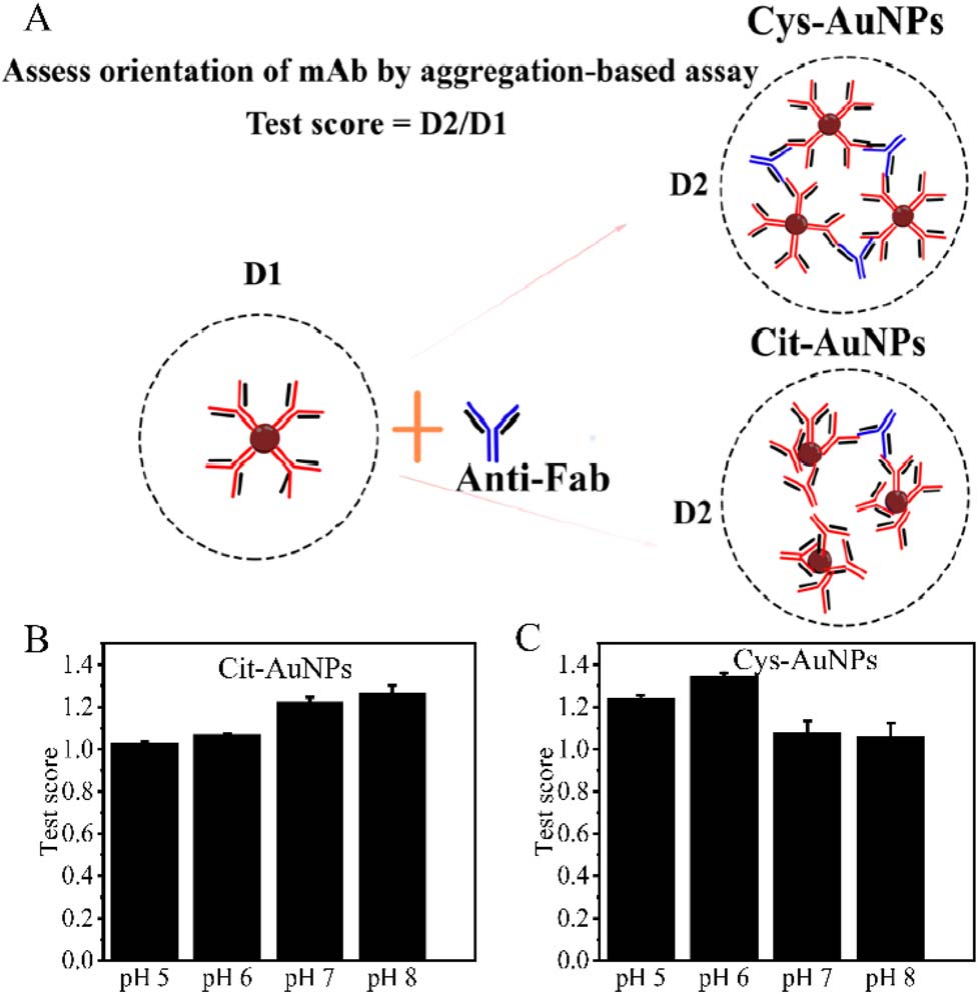
An aggregation-based assay to assess the orientation of mAb adsorbed onto AuNPs. (A) Illustration of the assay, showing the binding of anti-mouse IgG Fab-specific antibodies to the AuNP-mAb conjugates, leading to changes in hydrodynamic diameter. (B) Test scores (D2/D1 ratios) for Cit-AuNPs prepared at pH 5, 6, 7, and 8. The highest ratio, indicating optimal antibody orientation, is observed at pH 8. (C) Test scores (D2/D1 ratios) for Cys-AuNPs prepared at pH 5, 6, 7, and 8, showing the highest ratio at pH 6.

The orientation of the adsorbed antibodies on the AuNPs was further assessed using an aggregation-based assay, as described by Ruiz *et al.*^18^ In this assay, the hydrodynamic diameter of the conjugate between AuNPs and antibodies (D1) was measured using dynamic light scattering (DLS). Following this, anti-mouse IgG Fab-specific antibodies were added to the conjugate solution, and the hydrodynamic diameter was measured again (D2) using DLS. The addition of anti-Fab antibodies induces cross-linking among conjugates where the Fab fragments are oriented outward, resulting in a larger D2 value. A higher D2 value correlates with a greater number of Fab fragments that are accessible on the nanoparticle surface. The ratio of D2 to D1 (D2/D1) serves as a semiquantitative measure of aggregation and the accessibility of Fab fragments, as depicted in Fig. 5A. The results (Fig. 5B) revealed that the greatest aggregation, as indicated by the highest D2/D1 ratio, was observed for conjugates formed at pH 8 with Cit-AuNPs and at pH 6 with Cys-AuNPs. This indicates that Cys-AuNPs under acidic conditions (pH 6) provide a more favorable environment for antibody orientation, allowing more Fab antigen-binding sites to be accessible. Therefore, pH 8 for Cit-AuNPs and pH 6 for Cys-AuNPs were chosen as the optimal conditions for future experiments due to their superior ability to promote effective antibody conjugation and maximize aggregation. Table 2 provides a comparative analysis of dynamic light scattering (DLS) measurements, focusing on the hydrodynamic diameter of the conjugates post anti-Fab antibody addition (D2) and the ratio of D2 to D1 (D2/D1) for Cit-AuNPs and Cys-AuNPs. For Cit-AuNPs, the DLS measurement post-anti-Fab antibody addition (D2) is 65.30 ± 1.41 nm, with a D2/D1 ratio of 1.26 ± 0.04. In contrast, for Cys-AuNPs, the D2 value is markedly higher at 97.09 ± 1.69, and the D2/D1 ratio is significantly elevated at 1.34 ± 0.01 nm. These measurements indicate that Cys-AuNPs achieve a more substantial aggregation effect compared to Cit-AuNPs. The higher D2 and D2/D1 ratios for Cys-AuNPs suggest that these nanoparticles provide better exposure and accessibility of Fab fragments, particularly at an acidic pH of 6, enhancing the potential for cross-linking with anti-Fab antibodies. This observation aligns with the finding that pH 6 is optimal for Cys-AuNPs, as it fosters favorable antibody orientation, yielding more accessible antigen-binding sites.^29^

**Table 2:** DLS Comparison of anti-Fab (D2) and D2/D1 for Cit-AuNPs-LFS and Cys-AuNPs-LFS

Type of AuNPs	Characterization	Anti-Fab (D2)	D2/D1
Cit-AuNPs	DLS (nm)	65.30 ± 1.41	1.26 ± 0.04
Cys-AuNPs	DLS (nm)	97.09 ± 1.69	1.34 ± 0.01

### 3.3 Quantification of antibody conjugation efficiency for Cys-AuNP and Cit-AuNP

The conjugation efficiency of antibodies to Cys-AuNPs and Cit-AuNPs was assessed by determining the concentration of unbound antibodies remaining in the supernatant following centrifugation. This was achieved using a NanoDrop spectrophotometer. The results are presented in Table 3. Initially, 12 µL of mAb-NGAL at a concentration of 1.0 mg mL^−1^ was added to each type of AuNP, resulting in a total antibody input of 0.012 mg. After the conjugation process, the supernatant was collected, and the concentration of unbound antibodies was determined by measuring the absorbance at 280 nm using the NanoDrop. This procedure was performed in triplicate for each type of AuNP to ensure the accuracy and reproducibility of the conjugation efficiency results. For the Cys-AuNPs, the total amount of unbound antibodies in the 800 µL supernatant was calculated to be 0.00126 mg on average; for the Cit-AuNPs, the total unbound antibodies were found to be 0.004 mg on average. The total unbound antibody concentration was subtracted from the initial antibody concentration added during the conjugation process to determine the number of bound antibodies. This resulted in 0.01074 mg of bound antibodies for the Cys-AuNPs and 0.008 mg for the Cit-AuNPs. The percentage of bound antibodies was then calculated by dividing the number of bound antibodies by the initial amount of antibodies added and multiplying by 100. For Cys-AuNPs, the percentage of bound antibodies was approximately 89.54%, indicating a high conjugation efficiency. In contrast, Cit-AuNPs showed a lower conjugation efficiency, with about 66.67% of antibodies bound. These results demonstrate that Cys-AuNPs achieve a significantly higher antibody conjugation efficiency compared to Cit-AuNPs. The superior binding of antibodies to Cys-AuNPs can be attributed to the strong bonds formed between the cysteamine molecules and the gold surface, which provide a more stable attachment. On the other hand, Cit-AuNPs rely on weaker, non-covalent interactions, such as electrostatic forces, resulting in a lower binding efficiency. The high percentage of bound antibodies observed with Cys-AuNPs makes them a preferable choice for applications that require stable and durable antibody-nanoparticle conjugates, such as in diagnostic assays and biosensors.^30^ This enhanced stability and binding efficiency could lead to improved sensitivity and specificity in various analytical applications, including LFS.

**Table 3:** Antibody conjugation efficiency for Cys-AuNPs and Cit-AuNPs

AuNP type	Initial antibody (mg)	Unbound antibody (mg)	Bound antibody (mg)	% Bound antibody
Cys-AuNPs	0.012	0.00126	0.01074	89.54%
Cit-AuNPs	0.012	0.004	0.008	66.67%

### 3.4 Sensitivity for LFS using Cys-AuNPs and Cit-AuNPs

**Fig. 6:**
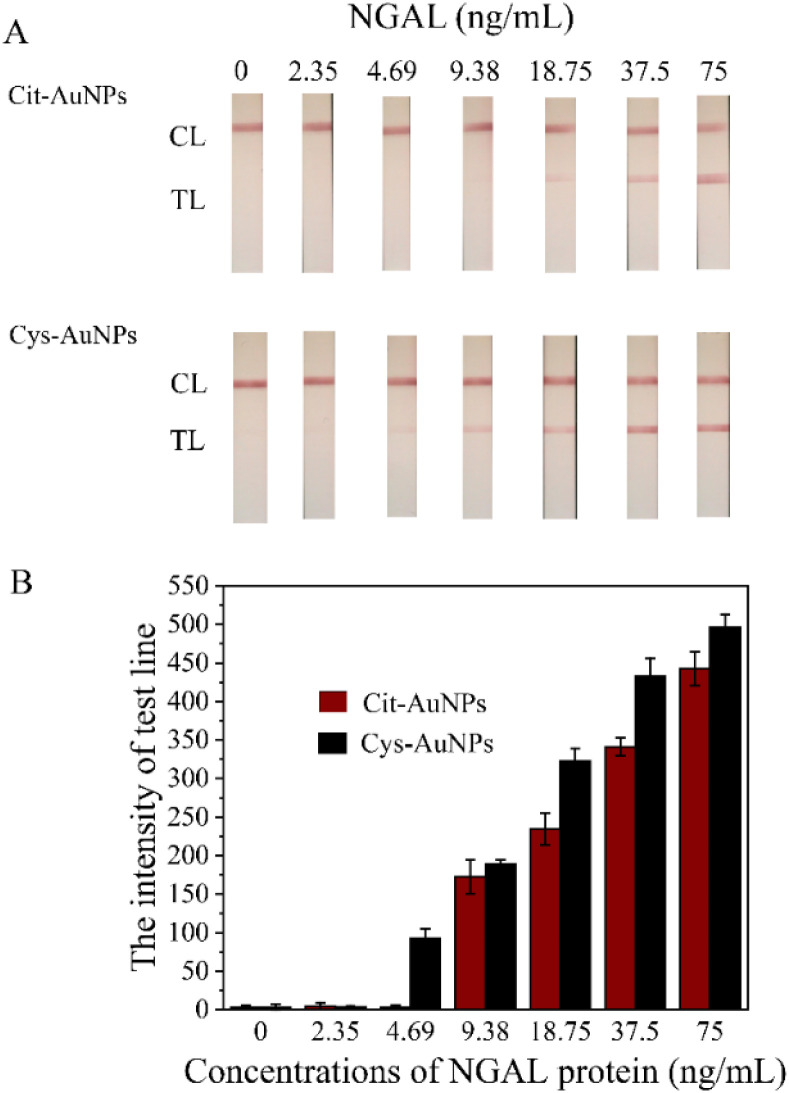
(A) Photographs of LFS test results using Cit-AuNPs-LFS and Cys-AuNPs-LFS for various NGAL concentrations (0, 2.35, 4.69, 9.38, 18.75, 37.5, and 75 ng mL^−1^). (B) Test-line intensity was measured by RapidScan ST5 LFS reader for standard NGAL concentrations (0, 2.35, 4.69, 9.38, 18.75, 37.5, and 75 ng mL^−1^), with averages from three data points and error bars for standard deviation.

Fig. 6 presents the analytical performance of the LFS using different types of AuNPs as labeling detectors. Fig. 6A shows the results of the LFS using Cit-AuNPs as the labeling detector. The visual lowest limit of detection (vLOD), defined as the lowest concentration detectable by the naked eye, was determined to be 18.75 ng mL^−1^ for this method. In contrast, Fig. 6B illustrates the results of the LFS using Cys-AuNPs as the labeling detector, where the vLOD was significantly lower at 4.69 ng mL^−1^, indicating enhanced sensitivity compared to the use of Cit-AuNPs. The improvement in sensitivity was further confirmed through intensity measurements using a strip reader, which showed that Cys-AuNPs provided a vLOD approximately four times lower than that achieved with Cit-AuNPs (from 18.75 ng mL^−1^ to 4.69 ng mL^−1^). This enhanced sensitivity is likely attributed to the positively charged surface of Cys-AuNPs, conferred by the terminal –NH_2_ groups of cysteamine, which promote favorable antibody orientation and increased binding efficiency. Antibodies possess negatively charged regions, particularly in the Fc (fragment crystallizable) domain, due to the presence of carboxylate (–COO^−^) groups and sialic acid residues.^28^ These negatively charged Fc regions are attracted to the cationic –NH_2_ groups on the Cys-AuNP surface through electrostatic interactions. As a result, the Fc portion binds preferentially to the nanoparticle, leaving the antigen-binding Fab (fragment antigen-binding) domains oriented outward and accessible to target NGAL molecules. This directional immobilization enhances antigen-binding capacity while preserving antibody functionality. In contrast, Cit-AuNPs carry a net negative surface charge, which may lead to random antibody adsorption *via* nonspecific interactions involving both Fc and Fab regions. This random orientation can reduce the accessibility of antigen-binding sites, thereby lowering detection sensitivity. Previous studies have demonstrated that positively charged nanoparticles facilitate more efficient antibody immobilization *via* Fc-region interactions, improving biosensor performance through enhanced signal strength and target recognition.^31,32^ Therefore, surface functionalization with cysteamine not only alters surface charge but plays a critical role in directing antibody orientation to achieve superior analytical performance.

As summarized in Table 4, most NGAL detection platforms reported to date rely on fluorescence or chemiluminescence-based immunoassays, such as the AlereTriage® NGAL Test and the ARCHITECT urine NGAL assay.^33,34^ While these methods provide reliable results with broad detection ranges, they require specialized instrumentation and are therefore less practical for decentralized or point-of-care testing.

**Table 4:** Comparison of NGAL detection platforms, methods, assay time, and detection range

Test name/platform	Method	Time to result	Detection range/vLOD (ng mL^−1^)	Company method
AlereTriage® NGAL test	Point-of-care fluorescence immunoassay	15 min	15–1300	BiositeInc/Alere
ARCHITECT urine NGAL	Chemiluminescent microparticle immunoassay (CMIA)	35 min	10–1500	Abbott diagnostics
NGAL rapid ELISA kit	Particle-enhanced turbidimetric immunoassay (PETIA)	10 min	25–5000	BioPorto diagnostics
NGAL ELISA kit	ELISA	1 h	2–2000	BioPorto diagnostics datasheet
Lipocalin-2/NGAL ELISA	ELISA	3.5 h	0.4–1000	Argutus medical datasheet
NGAL ELISA kit	ELISA	3.5 h	0.3–1000	BioVendor datasheet
This study (Cys-AuNPs LFS)	Lateral flow assay with cysteamine-stabilized AuNPs	∼10–15 min	3.5 (vLOD)	This work

ELISA-based platforms (BioPorto, Argutus, R&D/BioVendor) remain widely used due to their established performance in clinical research. They offer sensitive detection, with reported limits as low as 0.3–0.4 ng mL^−1^, but require long assay times (1–3.5 h) and laboratory infrastructure.^4^

Moreover, particle-enhanced turbidimetric immunoassays (PETIA) offer a faster turnaround time (10 min) and high throughput, but are limited to automated laboratory analyzers. In contrast, our Cys-AuNP-LFS achieves a visible limit of detection (vLOD) of 3.5 ng mL^−1^, which is comparable to the sensitivity reported for laboratory-based ELISA platforms while maintaining the speed and ease of use of rapid tests. Briefly, the average flow rate was approximately 6 mm min^−1^, and the total assay run time was 10–15 minutes. Importantly, unlike fluorescence- or chemiluminescence-based LFS, our approach does not require external readers, thereby improving accessibility for point-of-care applications. To the best of our knowledge, the use of Cys-AuNPs for NGAL detection in LFS has not been previously reported, representing a methodological advancement that enhances sensitivity while retaining the low-cost and user-friendly features of colorimetric LFS.

### 3.5 Evaluation of buffer conditions for conjugate pad preparation

**Fig. 7:**
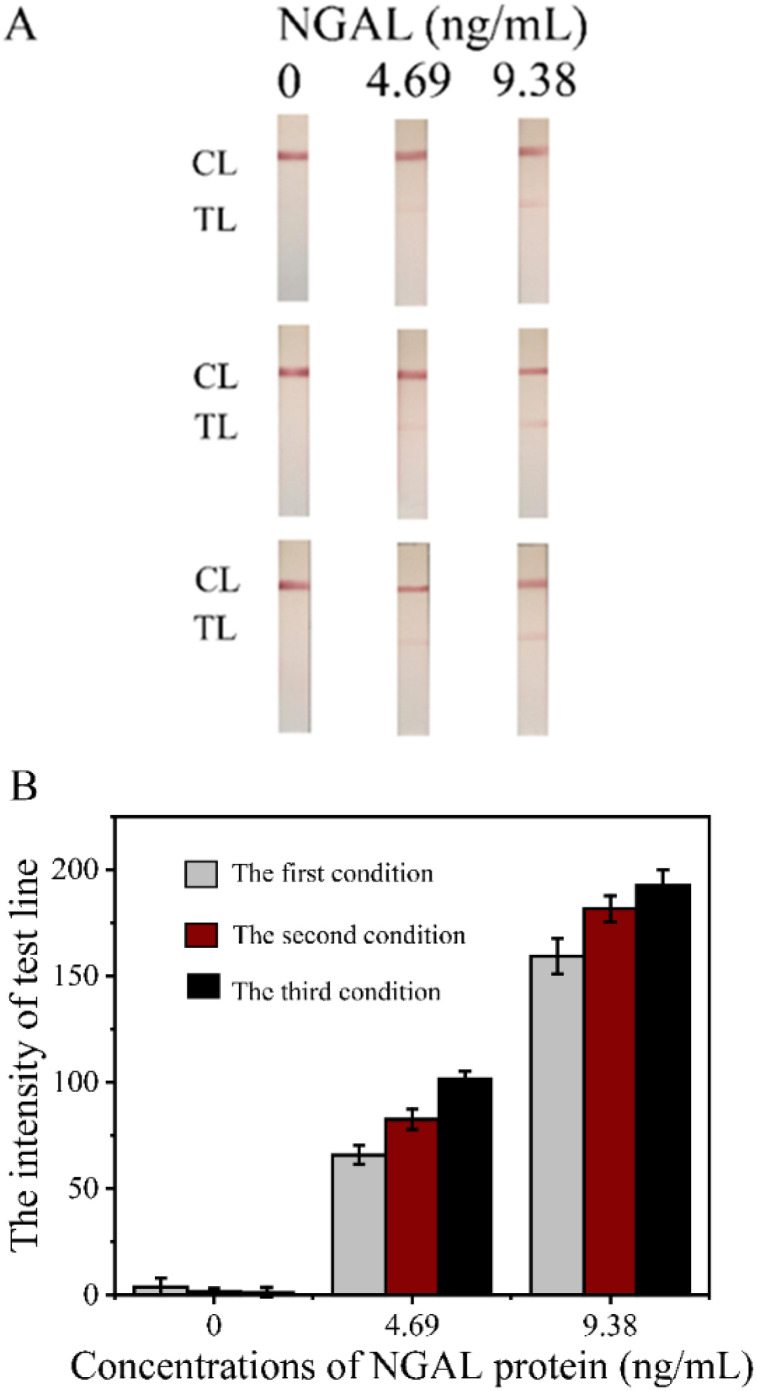
(A) Images of Cys-AuNPs-LFS under different buffer conditions: untreated pads (the first condition), phosphate buffer with 1% sucrose and 2% BSA (the second condition), and phosphate buffer with 5% sucrose, 1% BSA, and 0.25% Tween 20 (the third condition). (B) Test line intensity was measured by RapidScan ST5 LFS reader, with averages from three data points and error bars for standard deviation.

The results (Fig. 7) indicated that the third buffer condition, using the phosphate buffer supplemented with 5% sucrose, 1% BSA, and 0.25% Tween 20, produced the highest intensity of the test line. This suggests that this buffer formulation provided the best conditions for stabilizing the mAb-NGAL-AuNPs on the conjugate pads, likely due to an optimal balance of components that minimizes protein denaturation and enhances the release of the conjugate during the assay. In contrast, the first condition, where the pads were left untreated, resulted in the lowest intensity of the test line, indicating poor stability and release of the conjugate. The third buffer condition performed better than the second one due to its higher sucrose concentration (5% *versus* 1%). Sucrose is known to act as a stabilizing agent that protects proteins during drying by preventing denaturation and aggregation.^35^ The increased sucrose concentration in the third buffer likely provided a more protective environment for the mAb-NGAL-AuNPs during the drying process, resulting in better preservation of the antibody's functional structure and greater release efficiency during the assay. Additionally, the lower concentration of BSA (1% compared to 2% in the second condition) in the third buffer might have minimized potential steric hindrance or non-specific binding, further enhancing the stability and functionality of the conjugate.^36^ These findings demonstrate that the choice of buffer for pre-treating conjugate pads significantly impacts the performance of LFS strips. The third buffer condition proved to be the most effective in enhancing the signal intensity.

### 3.6 Specificity for LFS using Cys-AuNPs

**Fig. 8:**
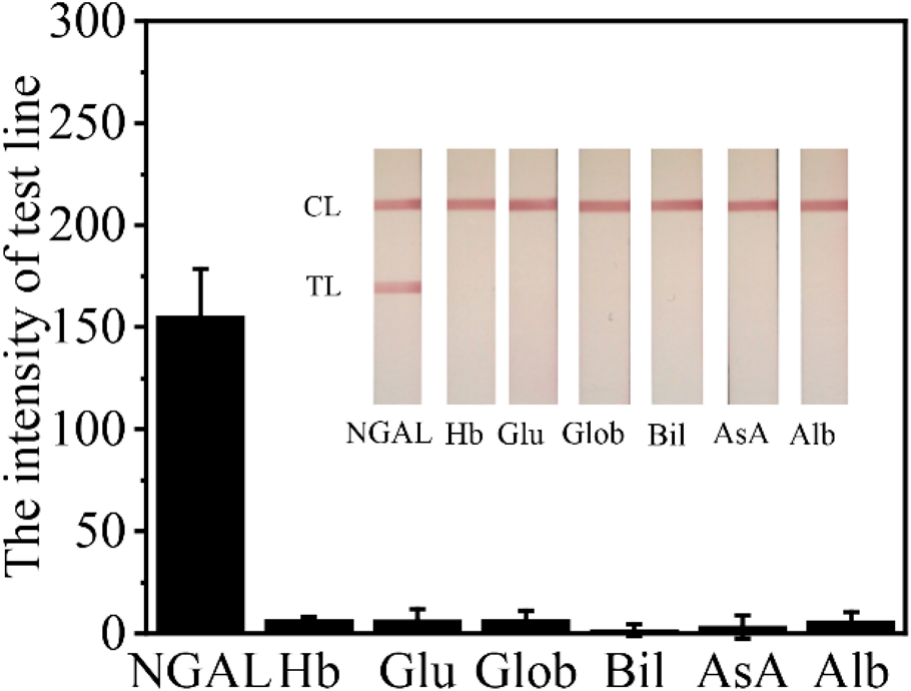
Analysis of specificity and test line intensity in LFS: substances evaluated include 9.38 ng mL^−1^ NGAL, 10 mg dL^−1^ hemoglobin (Hb), 500 mg dL^−1^ glucose (Glu), 10 mg dL^−1^ globulin (Glob), 4 mg dL^−1^ bilirubin (Bil), 40 mg dL^−1^ ascorbic acid (AsA), and 10 mg dL^−1^ albumin (Alb). Each data point represents the mean of three replicates, with error bars.

Several endogenous compounds commonly found in urine were tested to evaluate the specificity of LFS for detecting NGAL. Fig. 8 shows that the intensity data indicated no cross-reactivity with any of the other tested substances. This outcome demonstrates that the LFS exhibits specificity for NGAL, as the presence of other urine components did not interfere with the detection process. The lack of cross-reactivity suggests that the antibodies used in the LFS are highly selective for NGAL, ensuring accurate and reliable detection in complex biological samples. This high level of specificity is crucial for clinical applications, where precise identification of NGAL is necessary to avoid false positives and ensure the accuracy of diagnostic results.

### 3.7 Validation of LFS for NGAL detection in AKI diagnosis

**Fig. 9:**
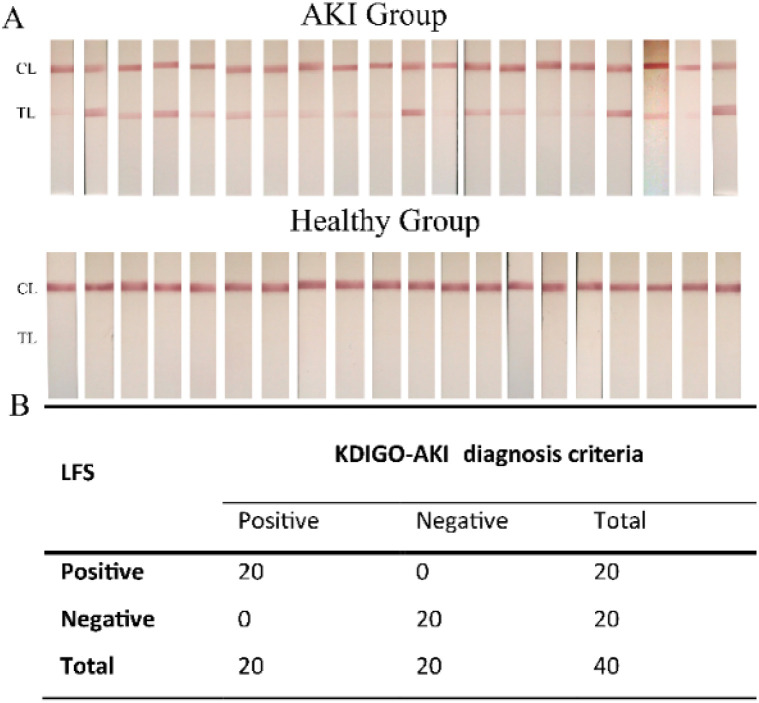
(A) Photographs of Lateral Flow Strips (LFS) for the Acute Kidney Injury (AKI) group (*n* = 20) and healthy controls (*n* = 20). (B) Tabular comparison between the results from the LFS and the Kidney Disease: Improving Global Outcomes (KDIGO) AKI diagnosis criteria for the AKI group (*n* = 20) and healthy controls (*n* = 20).

The performance of the LFS for NGAL detection in clinical urine samples was evaluated using specimens from AKI patients (*n* = 20) and healthy individuals (*n* = 20), classified based on KDIGO criteria.^37^ The results demonstrated that the LFS achieved 100% sensitivity and 100% specificity (Fig. 9), indicating its reliability and effectiveness for rapid NGAL screening. However, the study was limited by the small sample size.

### 3.8 Receiver operating characteristic (ROC) curve analysis of urine NGAL for AKI detection

**Fig. 10:**
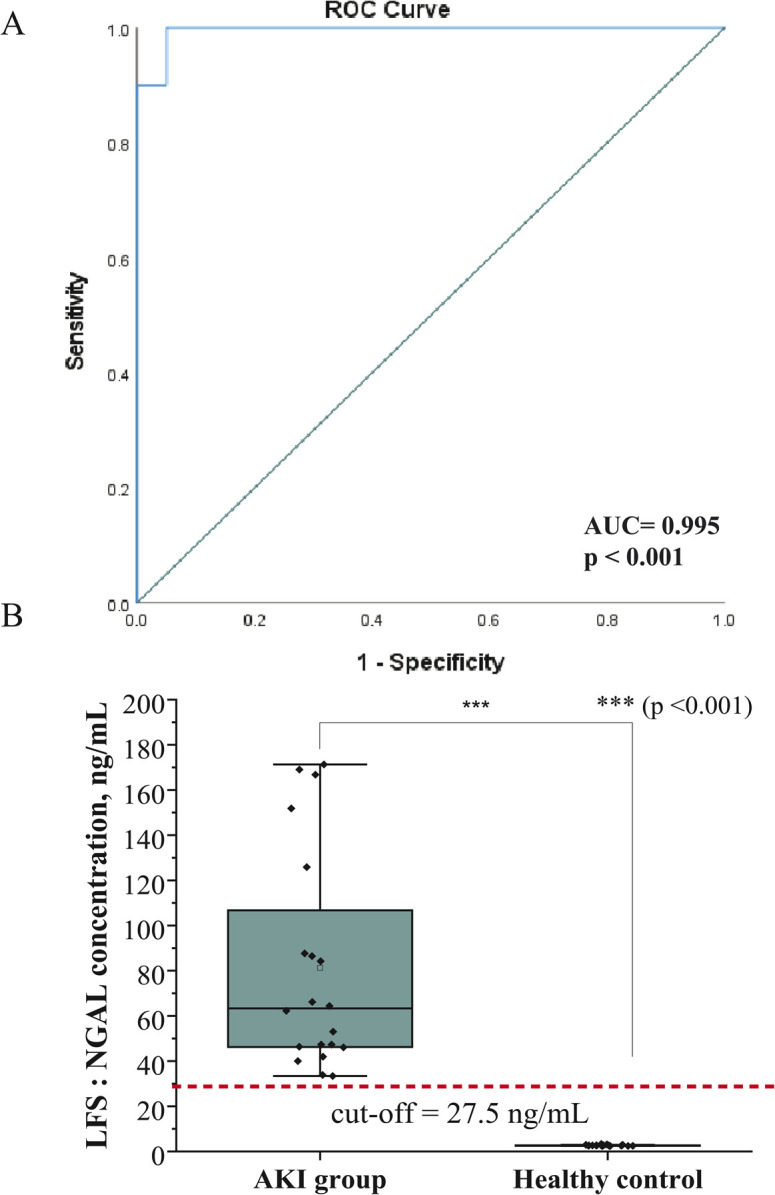
Evaluation of urine NGAL levels as a diagnostic biomarker for acute kidney injury (AKI) (A) Receiver Operating Characteristics (ROC) analysis of the LFS for detection of AKI in Urine. (B) Statistical comparison of median NGAL concentrations in AKI group (*n* = 20) and the healthy control (*n* = 20).

Our findings demonstrate that urine NGAL is a highly reliable biomarker for the early detection of AKI. ROC curve analysis produced an AUC of 0.995 (Fig. 10), clearly distinguishing AKI patients from healthy controls. Using a cutoff value of 27.5 ng mL^−1^, the assay achieved a sensitivity of 100% and a specificity of 95%. This performance indicates that the selected threshold could serve as a robust and clinically useful indicator for AKI screening. Notably, our cutoff is lower than the 50 ng mL^−1^ threshold reported in some earlier studies, suggesting that our approach may provide improved sensitivity and allow for the detection of kidney injury at an earlier stage. Previous reports have emphasized the diagnostic utility of NGAL as an early biomarker of AKI, as urine NGAL levels strongly correlate with the severity of renal injury and can effectively discriminate AKI from non-AKI patients.^38^ Our study adds to this evidence by highlighting the advantage of a lower cutoff value, which may enhance early detection without significantly reducing specificity. Overall, these results support the potential of NGAL LFS-based assays as rapid, non-invasive, and highly sensitive tools for AKI diagnosis. Nevertheless, NGAL elevations are not exclusive to AKI and may also be observed in CKD, sepsis, or systemic inflammation, which can compromise specificity in some settings.^39^ To confirm the broader clinical applicability of this cutoff, further validation in larger and more diverse cohorts is needed.

### 3.9 Reproducibility

**Fig. 11:**
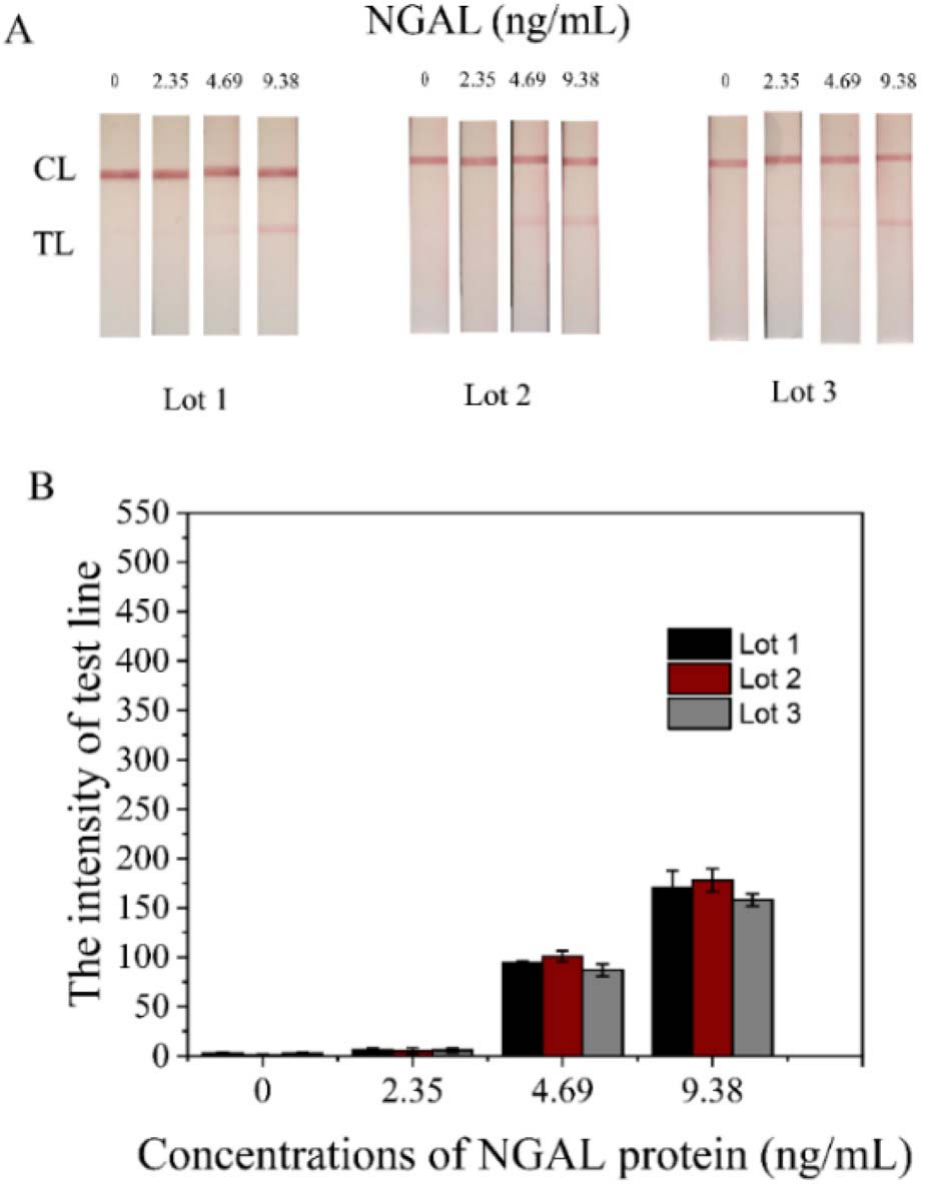
Reproducibility and performance of the developed NGAL-LFS method. (A) Representative lateral flow strip (LFS) images tested with NGAL at concentrations of 0, 2.35, 4.69, and 9.38 ng mL^−1^, demonstrating the concentration-dependent increase in test line intensity. (B) The coefficient of variation (CV) for reproducibility was calculated across three independent production days, yielding an average CV of 3.17%.

The average coefficient of variation (CV) for reproducibility was 3.17%. This was determined by evaluating the method's consistency across three separate production days (Fig. 11). A CV of 3.17% indicates that a method demonstrates good reliability and is capable of generating consistent results over multiple production days. CVs below 5% are considered indicative of high precision and low variability between assay runs.^39^

### 3.10 Stability evaluation of conjugated-Cys-AuNPs in solution and dry storage conditions

**Fig. 12:**
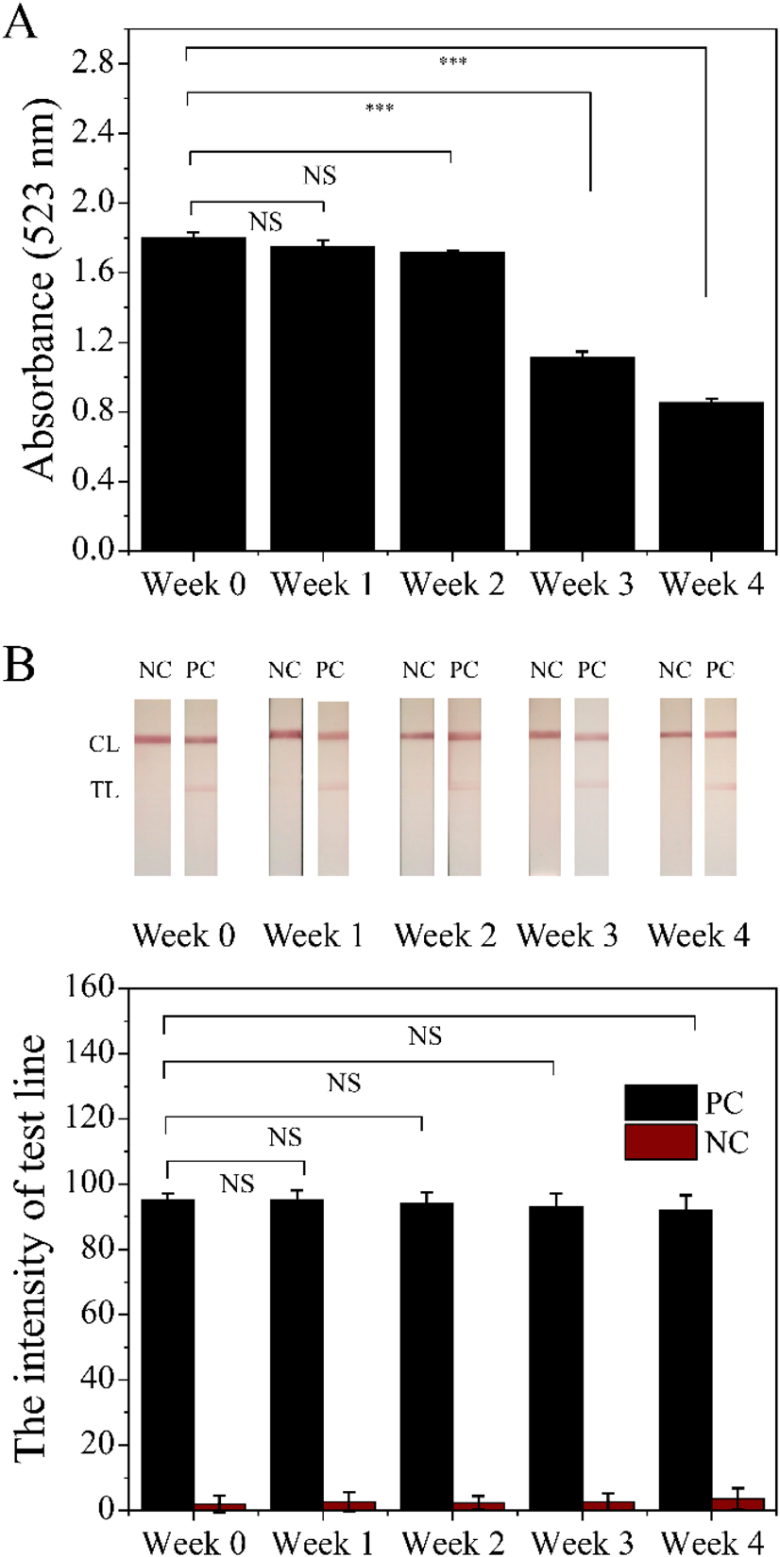
Assessment of the stability of Conjugated Cys-AuNPs (A) conjugates stored in solution monitored by UV-Vis absorbance spectroscopy. (B) Conjugates dried on the conjugate pad of lateral flow strips demonstrated maintained test line intensity over four weeks. The analysis includes positive control (PC) and negative control (NC) samples, where PC corresponds to NGAL 4.69 ng mL^−1^ and NC corresponds to NGAL 0 mg mL^−1^. Each number represents the average of three data points, with error bars indicating standard deviations. (NS, not-significant; ***p < 0.001).

The stability of conjugated-Cys-AuNPs was assessed over four weeks under two different storage conditions: (1) conjugated-Cys-AuNPs stored in solution and (2) conjugated-Cys-AuNPs dried onto conjugate pads in LFS. To evaluate the stability of the conjugated-Cys-AuNPs in solution, 1% (w/v) NaCl was added weekly to each tube, and the absorbance was measured using UV-Vis spectroscopy. For the dried conjugates, stability was monitored by measuring the test line intensity on LFS at each time point. The results (Fig. 12) showed that the conjugated-Cys-AuNPs stored in the solution exhibited significant changes over time, while those in the dry state remained relatively stable. The UV-Vis absorbance spectra of the solution-stored conjugates showed a gradual decrease in peak intensity and broadening, indicating increased aggregation over time. These changes were more pronounced after the second week, suggesting a loss of stability due to salt-induced aggregation. In contrast, the dried conjugates exhibited minimal changes, with the test line intensity on LFS remaining relatively stable across the four weeks. This indicates that the dried conjugates maintained their performance more effectively than the solution-stored conjugates. The differences in stability between the two storage conditions can be attributed to the protective effect of the dried matrix. In solution, nanoparticles are more susceptible to environmental stressors such as ionic strength fluctuations, leading to aggregation and reduced stability. However, in the dry state, the conjugates are stabilized within the conjugate pad matrix, which helps preserve their integrity and prevents nanoparticle aggregation. These findings suggest that storing Cys-AuNP conjugates in a dry format may enhance their long-term stability compared to storage in a solution.

## 4 Conclusions

This study demonstrates the superior performance of Cys-AuNPs over traditional Cit-AuNPs as detection labels in LFS for NGAL detection. The Cys-AuNPs exhibited enhanced binding affinity and signal intensity, significantly improving sensitivity and lower detection limits. The findings underscore the potential of Cys-AuNPs to enhance the performance of LFS, making them more effective tools for rapid and accurate point-of-care testing. This improvement is particularly crucial for the early diagnosis of acute kidney injury (AKI), where timely detection of NGAL can lead to better patient outcomes through early intervention. Furthermore, the successful application of Cys-AuNPs in this study highlights their potential for broader use in biomarker detection, expanding the impact of this novel labeling approach across various diagnostic contexts. However, a limitation of conjugated Cys-AuNPs is their stability in solution, as they tend to become unstable after two weeks. Nevertheless, their stability improves significantly when conjugates are dried on the conjugate pad. Optimizing the formulation to address this limitation could further improve their stability and enhance their practicality in diagnostic applications. In conclusion, the integration of Cys-AuNPs in LFS represents a significant advancement in diagnostic technology, providing a more sensitive, reliable, and user-friendly platform for biomarker detection.

## Ethical statement

All experiments involving human urine samples were conducted in accordance with the Declaration of Helsinki, the “Clinical Investigation of Medical Devices for Human Subjects – Good Clinical Practice and the National Research Council of Thailand's Guidelines for Research Involving Human Participants”. The study protocol was reviewed and approved by the Khon Kaen University Ethics Committee for Human Research (Institutional Review Board Number: HE684002). Human urine samples used in this study were anonymized leftover specimens obtained from routine clinical laboratory testing (Srinagarind Hospital, Khon Kaen University, Thailand). Therefore, informed consent was waived by the Ethics Committee in accordance with institutional and national ethical guidelines.

## Author contributions

Conceptualization, P. T. (Paweena Tunakhun), J. D., S. D., and P. B.; methodology, P. T. (Paweena Tunakhun), P. M., J. P., and N. C.; investigation, J. D.; P. B.; K. C.; P. T. (Patcharaporn Tippayawat); R. T.; and S. A. resources, S. A.; data curation, P. T. (Paweena Tunakhun), and J. D.; writing – original draft preparation, P. T. (Paweena Tunakhun), S. N., and J. D.; writing – review and editing, P. T. (Paweena Tunakhun), J. D., S. N., O. S, and P. B.; supervision, J. D., P. T. (Paweena Tunakhun), and P. B., all authors have read and agreed to the published version of the manuscript.

## Conflicts of interest

There are no conflicts to declare.

## Data Availability

No primary research results, software or code have been included and no new data were generated or analysed as part of this review.
